# Nanocellulose Reinforced Thermoplastic Starch (TPS), Polylactic Acid (PLA), and Polybutylene Succinate (PBS) for Food Packaging Applications

**DOI:** 10.3389/fchem.2020.00213

**Published:** 2020-04-15

**Authors:** A. Nazrin, S. M. Sapuan, M. Y. M. Zuhri, R. A. Ilyas, R. Syafiq, S. F. K. Sherwani

**Affiliations:** ^1^Laboratory of Biocomposite Technology, Institute of Tropical Forestry and Forest Products, Universiti Putra Malaysia, Seri Kembangan, Malaysia; ^2^Advanced Engineering Materials and Composites Research Centre (AEMC), Department of Mechanical and Manufacturing Engineering, Universiti Putra Malaysia, Seri Kembangan, Malaysia

**Keywords:** food packaging, nanocellulose, polybutylene succinate (PBS), polylactic acid, thermoplastic starch

## Abstract

Synthetic plastics are severely detrimental to the environment because non-biodegradable plastics do not degrade for hundreds of years. Nowadays, these plastics are very commonly used for food packaging. To overcome this problem, food packaging materials should be substituted with “green” or environmentally friendly materials, normally in the form of natural fiber reinforced biopolymer composites. Thermoplastic starch (TPS), polylactic acid (PLA) and polybutylene succinate (PBS) were chosen for the substitution, because of their availability, biodegradability, and good food contact properties. Plasticizer (glycerol) was used to modify the starch, such as TPS under a heating condition, which improved its processability. TPS films are sensitive to moisture and their mechanical properties are generally not suitable for food packaging if used alone, while PLA and PBS have a low oxygen barrier but good mechanical properties and processability. In general, TPS, PLA, and PBS need to be modified for food packaging requirements. Natural fibers are often incorporated as reinforcements into TPS, PLA, and PBS to overcome their weaknesses. Natural fibers are normally used in the form of fibers, fillers, celluloses, and nanocelluloses, but the focus of this paper is on nanocellulose. Nanocellulose reinforced polymer composites demonstrate an improvement in mechanical, barrier, and thermal properties. The addition of compatibilizer as a coupling agent promotes a fine dispersion of nanocelluloses in polymer. Additionally, nanocellulose and TPS are also mixed with PLA and PBS because they are costly, despite having commendable properties. Starch and natural fibers are utilized as fillers because they are abundant, cheap and biodegradable.

## Introduction

The use of non-degradable plastics lead to global warming, water pollution, and air pollution (Abral et al., 2019b). The average annual world plastics production has been steadily increasing from 270 million tons per year in 2010 to 359 million tons per year in 2018, or about 33% globally (Garside, 2019), with 62 million metric tons produced in Europe alone. China is one of the largest producers of plastics in the world, accounting for more than one quarter of the global production. At the end of their life cycle, these plastics become solid waste. They will pollute the environment for a long time as they do not degrade or break down in natural environments. Furthermore, the manufacturing of synthetic polymers and disposal through incineration produces CO_2_ and thus contributes to global warming (Dorgan et al., 2001). Synthetic polymers or oil derivative polymers have a direct impact on the environment. To overcome these problems, researchers are mainly focused on renewable resources, which are biodegradable (Halimatul et al., 2019; Ilyas and Sapuan, 2020). For packaging materials, researchers have avoided the use of non-degradable plastics and focus on renewable and biodegradable resources (Muller et al., 2017). Synthetic plastics are made from a petroleum-based polymer material and are the most commonly used plastics today. The most practical solution for piles of plastic waste depends on the biodegradable material resources used to manufacture a product. If current plastics are made from fossil-based materials, the next plastic can be produced from biodegradable materials (Jumaidin et al., 2019a,b). The world's population is expected to grow to 9 billion by 2050 and this will further increase the production of plastics. Certainly, this will increase plastic waste (Emadian et al., 2017). Bioplastics, such as polylactide (PLA), poly butylene succinate (PBS), and thermoplastic starch, have therefore caught people's attention (Su et al., 2019). Starch is cheap and easily available in comparison to PLA. Starch-based polymer exhibits low mechanical and barrier properties (Syafri et al., 2019), while, PLA and PBS exhibit high mechanical and barrier properties despite their high cost and limited stock. However, synthetic plastics display superior properties compared to their bioplastic counterparts.

To catch up with synthetic plastic, biomaterials are formulated into biocomposite materials to create bioplastics which are comparable to synthetic plastic. Natural fiber is also used as a filler to reinforce the bioplastic's mechanical and barrier properties (Ilyas et al., 2019a). The combination of these materials has created promising biocomposite products, which are safe, biodegradable, and sustainable (Abral et al., 2019a). PLA and PBS, which are costly, are mixed with starch and natural fiber to achieve stable viable products. Starch and natural fiber are known for their abundance, low cost, biodegradable, and sustainable characteristics which are often used to enhance the properties of PLA and PBS. Even though starch is immiscible with PLA and PBS, the plasticization of starch has improved the interfacial bonding into the polymer matrix (Wang et al., 2008). The excellent properties of the materials are preserved, and inferior properties are offset by each other. Since all the materials used originate from plants, the disposal of plastic waste is manageable, while the decomposing process—which takes centuries—is reduced to several weeks.

Furthermore, the factor that was prioritized in the utilization of natural fiber to enhance polymer composites decreased reliance on timber by increasing the source of raw materials (natural fibers) to some degree (Sanyang et al., 2016b, 2018; Ilyas et al., 2018a,b,c, 2019c; Sapuan et al., 2018). Moreover, by enhancing the composites with natural fiber it helps the partial decomposition of unused products; hence partly solving the environmental issues (Liu et al., 2017). Unwanted natural fiber composites can be reused by crushing them into smaller sizes and remaking them into different new forms. The idea of using natural fibers as an enhancement in composites is not complete because of its outstanding fiber properties, but also on its impact to the exploitation of plant waste, preservation of forestry, biodiversity and obtainability of fiber plentifully at a low price. Furthermore, natural fibers can be obtained from by-products of agriculture and forestry, such as rice straws, corns, pineapples, oil palm and sugar palm fibers (Ilyas et al., 2018e). Besides, a more unique feature of fiber called nanocellulose can be extracted by using chemical acid hydrolysis process. Nanocellulose is another promising material that acts as a reinforcement filler for composite materials for its lightweight, high surface area, high mechanical strength, high aspect ratio and low density (Ilyas et al., 2018f). Low-cost, abundant and environmental-friendly materials are utilized to produce composite for single-used items (Ilyas et al., 2017, 2018d,f, 2019b).

## Nanocellulose Reinforced TPS Biopolymer Composites

**Figure 1:**
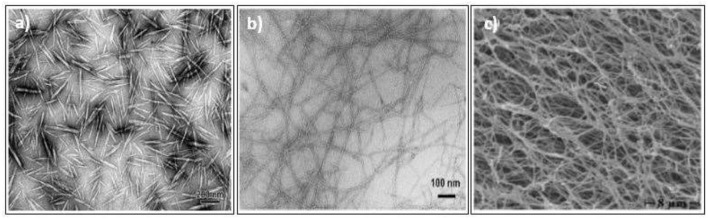
Comparison microscopic image of; **(a)** nanocrystalline cellulose (NCC), **(b)** nanofibrillated cellulose (NFC), **(c)** bacterial nanocellulose (BNC) (adapted, with permission from Ilyas et al., 2018e).

Nanocelluloses can be divided into three main categories; nanocrystalline cellulose (NCC), nanofibrillated cellulose (NFC), and bacterial nanocellulose (BNC) (Ilyas et al., 2018e) (Figure 1). Both NCC and NFC can be produced through the isolation and extraction of the by-products of agriculture and forestry using chemical, physical, and enzymatic processes. Meanwhile, BNC cultivated from bacteria (*Glueconoacetobackerxylinius*) in microfibrils are used as the medium. NCC was proven to decrease the water vapor permeability of starch-based films by improving the tortuosity of the films (Ilyas et al., 2018c). The high compatibility of the starch matrix and nanocellulose fillers enhances the film water sensitivity which makes a decent feature for food packaging applications.

Ilyas et al. (2020) mentioned that NCC, which acted as a reinforcing filler for starch-based polymers, was ideal due to the presence of abundant surface hydroxyl groups on the NCC surface which are responsible for hydrogen bonding between the non-polar matrices and –OH groups of hydrophilic polymer matrices. High aspect ratio and mechanical strength have shown interest in using NCC as a load-bearing tool in engineering new and affordable biodegradable materials (Mishra et al., 2018).

Ilyas et al. (2018b) developed a sugar palm nanocrystalline cellulose (SPNCC) reinforced with sugar palm starch (SPS) bionanocomposites for food packaging application. The Field Emission Scanning Electron Microscope (FESEM) image showed pure SPS films had a smooth surface while SPS/SPNCC films had a rough surface, but a lower content of SPNCC in SPS/SPNCC films had a much smoother surface as compared to a higher content. The rough surface feature was the result of low interfacial bonding between SPS and SPNCC. With lower content of SPNCC in SPS/SPNCC films, there were no visible agglomeration and clusters, suggesting that a finely homogenous dispersion of SPNCC was in the SPS matrix. Furthermore, SPS and SPNCC have the same botanical origin with similar chemical composition and hydrogen bond links between the nano-sized filler and matrix. Fine phase dispersion and strong adhesion of the nano-sized filler in the matrix were presumed to improve the mechanical properties of bionanocomposite films.

In another work, Ilyas et al. (2018f) studied the potential applications of NCC reinforced bioplastic polymer composites. In packaging application, NCC reinforced polymers enhanced their mechanical properties, thermal stability, and electrical conductivity, resulting in good barrier properties (Bagde and Nadanathangam, 2019). The substitution of high density glass fibers with low density NCC fillers produced lightweight materials which can be used in automotive applications to reduce fuel consumption by any means of reducing the CO_2_ gas emission (Mondal, 2017). NCC which was revealed to be harmless to humans and animal bodies is used for fluorescence bioimaging, bioassay and drug delivery applications, which can lower the cost for medical treatment (Ilyas et al., 2018e).

**Figure 2:**
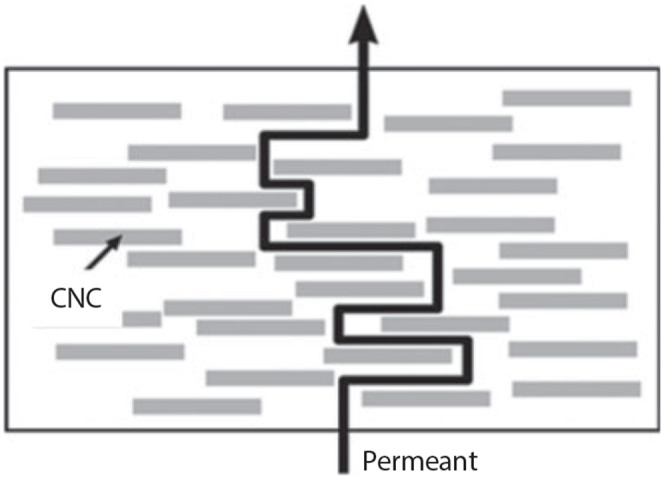
Schematic drawing of gas permeant diffusion through the structure of NCC incorporated polymer bionanocomposites (adapted, with permission from Criado et al., 2018).

Criado et al. (2018) studied the effect of NCC reinforcing biopolymer films for food packaging. Poor barrier and thermo-mechanical properties of biopolymers were unsuitable in the commercial packaging industry, which led to the improvement of the material by incorporating NCC as the filler. Aside from the excellent mechanical properties as a reinforcing agent, the incorporation of NCC into biopolymer films can lower the water vapor permeability rate (Figure 2). Furthermore, the bioactive compound in the biopolymers can be stabilized using NCC by controlling only an allowable amount to be released into the food. The development of bionanocomposite films incorporated NCC is able to improve food quality, prolong food shelf-life, and provide resistance against microbes, fungal and insect infections.

(Bagde and Nadanathangam, 2019) reported their findings on the antibacterial, biodegradable, and mechanical properties of corn starch films infused with bacteriocins immobilized on the surface of NCC. Two bacteriocins used were extracted from *P. acidiclactic* and *E. faecium* and NCC was prepared by bio-medical process using cotton linters. At room temperature, the bacteriocin immobilized NCC (BIN) films remained fresh for 4 weeks, while that of bacteriocin incorporated films degraded in 2 weeks. NCC films had the highest degradation rate, followed by BIN and bacteriocin films. The water solubility increased with the incorporation of NCC and BIN films by 36 and 41–46%, respectively. The tensile strength of NCC incorporated films and BIN significantly increased by 6 and 39–69%. There is great potential for BIN incorporated starch films to be utilized in food packaging.

**Figure 3:**
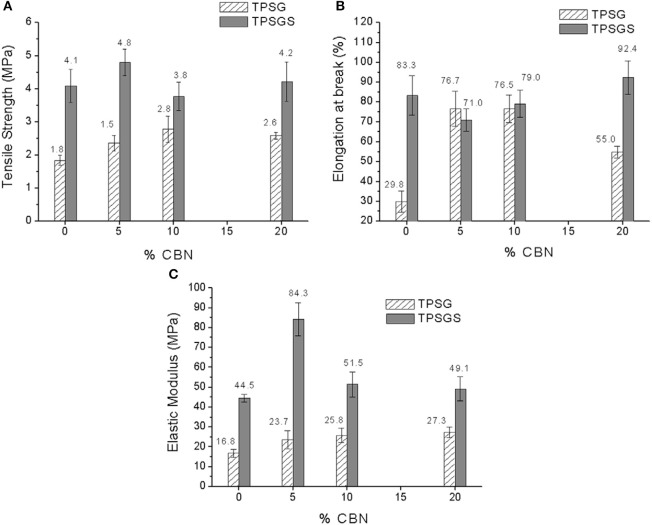
The mechanical properties TPS/CBN bionanocomposites **(A)** tensile strength (MPA); **(B)** Elongation at break (%); and **(C)** Elastic Modulus (MPA) (adapted with permission from Teixeira et al., 2009).

Teixeira et al. (2009) developed cassava nanofibrillated cellulose (CBN) incorporated thermoplastic cassava starch by melt mixing and hot pressing. The samples were prepared with different compositions of plasticizers (glycerol and sorbitol) infused CBN. The combination of both plasticizers (1:1) resulted in better tensile strength and elastic modulus as compared to only glycerol (Figure 3). The addition of CBN into TPS increased the water uptake which greatly affected the glycerol plasticized samples. The increasing CBN loading did not essentially increase the tensile strength and elastic modulus but was favorable for water uptake because of the hydrophobicity properties of CBN. The glycerol/sorbitol mixture hindered the good matrix bonding of CBN due to the transcrystallization of amylopectin on CBN surface.

Montero et al. (2017) investigated the reinforcement of NCC into thermoplastic starch (TPS) films from different vegetal sources for potential short shelf-life packaging applications. Thermoplastic pea starch (TPeS) with 35% of amylose formed a rigid amylose network with gelatinized granules. Rich amylopectin starch like thermoplastic potato starch (TPoS) and thermoplastic corn starch (TCS) demonstrated good water resistance and thermal stability but poor stiffness. The incorporation of NCC shifted the degradation starting temperature to a higher state. TPoS films resisted water the most with low diffusivity as compared to the other two. Regardless of resources, the increment of NCC loading decreased the water absorption rate due to the good interfacial bonding between nano-sized filler and starch matrix. Based on the results, TPoS can be a potential alternative for short shelf-life packaging applications.

## Nanocellulose Reinforced PLA Biocomposites

Generally, nanocellulose reinforcing PLA biocomposites contribute to the improvement of tensile strength and elastic modulus. The hydrophilic nanocellulose and hydrophobic PLA are incompatible, forming a weak matrix bonding which is the reason why only minimal nanocellulose loadings between 0.5% and 2 wt.% are required for optimal results (Kargarzadeh et al., 2018). To improve the compatibility and dispersion state of PLA/nanocellulose, surface characteristics of nanocellulose will undergo some chemical and mechanical modifications.

(Wang and Drzal, 2012) performed a different approach to hinder the poor compatibility and dispersion phase of nanocellulose into PLA using microencapsulation mixing and compression molding. PLA was dissolved using a solvent evaporation method, mixed with NCC under high-pressure homogenizer. Water was removed using membrane filtration-created PLA that contained nanocellulose sheets. Accumulated sheets were pressed using compression molding, producing PLA/nanocellulose composites. The reinforcement of NCC into the PLA matrix improved the modulus and strength (up to 58 and 210%, respectively) of the composites.

**Figure 4:**
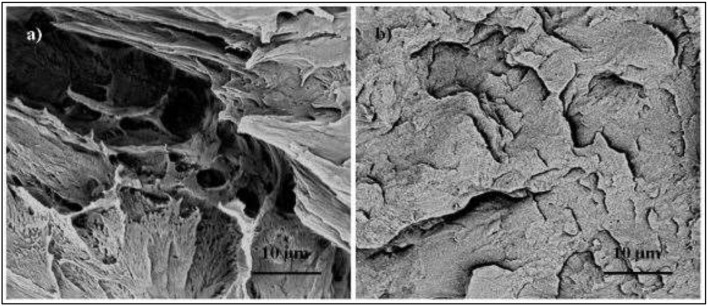
Cryo-fracture surface images using scanning electron micrographs test; **(a)** PLA and **(b)** PLA/NCC (3 wt.% of NCC) (adapted, with permission from Sullivan et al., 2015).

Sullivan et al. (2015) prepared PLA/NCC bionanocomposite films *via* melt blending and compression molding at 175°C for 3 min. The PLA/NCC mixture underwent melt spinning fiber before the compression molding process to reduce the agglomeration of NCC and improved the distribution of NCC along the fiber long axis. In Figure 4 the SEM images show that the bionanocomposites films have more surface fracture events as compared to pure PLA, which indicated that the incorporation of NCC contributed to a more brittle PLA. By increasing the amount of NCC, it further increased the film's degree of crystallinity due to the crystallization with CNN during the extended time of the slow compression molding cooling rate. Overall, NCC acted as nucleating agents by increasing the polymer matrix crystallinity, improving the crystallization and more brittle properties.

Trifol et al. (2016) prepared hybrid nanocomposite films by reinforcing pure PLA with nanocellulose, either nanofibrillated cellulose (NFC) or nanocrystalline cellulose (NCC), and nanoclay (Cloisite30B). Their barrier properties and thermomechanical resistance were investigated with 1 or 5 wt.% of nanocellulose incorporated with 1 wt.%, 3 wt.%, 5 wt.% of nanoclay. The combination of PLA with both nanocellulose and nanoclay resulted in a decreased oxygen transmission rate (OTR) to 90% and water vapor transmission rate (WVTR) to 76%. The thermomechanical resistance and crystallization kinetics were improved while their high transparency was maintained.

Khoo et al. (2016) studied the morphological and thermal properties of PLA/nanocellulose bionanocomposite films prepared using solution casting. PLA was incorporated with 1, 2, and 5 wt.% of nanocrystalline cellulose. The NCC incorporation accelerated the crystallization of PLA, proven by PLA/NCC-5, which showed high crystallinity (34.5%) as compared to pure PLA (30.9%). The decomposition temperature of the films increased as the NCC loading increased, which showed that NCC improved the thermal stability and slowed down the thermal degradation rate.

Hegyesi et al. (2019) tested the degradation rate of PLA/NCC bionanocomposites by using enzymatic agents, namely lipase from *Candida rugosa* (CRL) and protease (proteinase K from *Tritirachium album*). Proteinase K catalyzed the degradation rate of PLA/NCC sample while CRL did not. However, the drop in pH value to 4 denatured the enzyme which halted the degradation process. Additionally, increasing the ion concentration decreased the lost weight of the sample upon attaining equilibrium, which indicated that larger ionic strength slowed down the enzyme's activity. The reinforcement of NCC into PLA rapidly increased the degradation rate, by which the samples started to degrade in 3 days.

**Figure 5:**
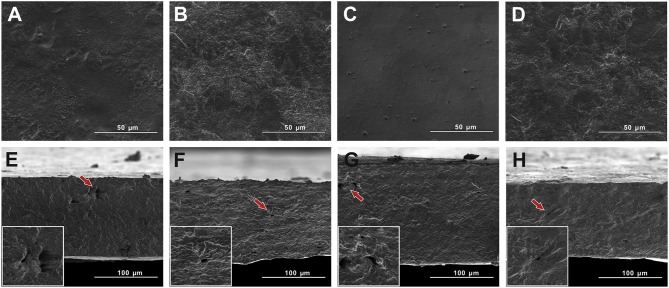
The images from SEM analysis showed the surface (**A**: SL, **B**: SLC, **C**: SLE, **D**: SLEC) and cross sections (**E**: SL, **F**: SLC, **G**: SLE, **H**: SLEC). The cross sections showed that nanocellulose reinforcement into PLA **(F,H)** have smaller pores compared to without nanocellulose **(E,G)** (adapted, with permission from Yu et al., 2019).

Yu et al. (2019) developed food packaging materials using NFC and *Cedrus deodara* pine needle extract (PNE) reinforcing PLA nanocomposite films (Figure 5). Four different composition samples were prepared pure: PLA (SL), PLA/NFC (SLC), PLA/PNE (SLE), and PLA/NFC/PNE (SLEC). The addition of NFC into PLA increased the tensile strength due to the filling properties of NFC. Furthermore, PNE incorporated films showed an inhibitory effect on food-borne bacteria which improved the antimicrobial properties of the films. The light barrier properties of the films were improved with the combination of NFC and PNE. These findings demonstrated that nanocomposite films exhibited commendable properties as a potential green food packaging.

**Figure 6:**
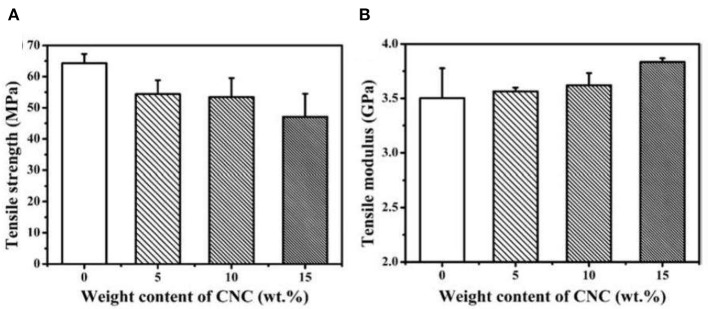
Tensile properties of PLA/NCC, **(A)** tensile strength; **(B)** tensile modulus (adapted, with permission from Zhang et al., 2019b).

**Figure 7:**
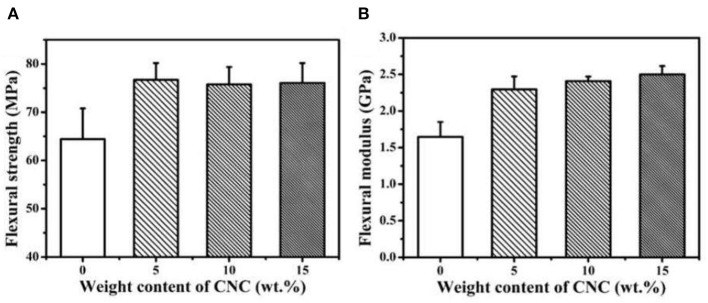
Flexural properties of PLA/NCC, **(A)** flexural strength (MPa); **(B)** flexural modulus (GPa) (adapted, with permission from Zhang et al., 2019b).

Zhang et al. (2019b) studied the mechanical, rheological and thermal properties of PLA/nanocellulose bionanocomposite films fabricated using Pickering emulsion. It showed that NCC loading improved the transition of films from liquid to solid-like state at high temperature. The onset crystallization temperature increased, which showed that the addition of NCC as a nucleating agent promoted polymer chain kinetics. The onset temperature of thermal decomposition also increased as NCC was added. This could be related to the char formation of NCC in the composites which slowed the thermal decomposition of the PLA matrix down. Figures 6, 7 show that the flexural and tensile modulus increased as the NCC loading increased due to the good dispersion of NCC in the PLA matrix. The Pickering emulsion approach promoted a more homogenous dispersion of NCC that enhanced significant properties of PLA films.

### PLA/TPS Blend Biocomposites

The incorporation between PLA/TPS and compatibilizer was performed to improve the interfacial interaction between both biopolymers (Soares et al., 2013). Several authors have added trimethoxy silane coupling agents (3-chloropropyl trimethoxy silane), anhydride functionalized polyester, citric acid (CA), formamide, maleic anhydride (MA), epoxidized itaconic acid (EIA), bio-based ether epoxidized cardanol (Epicard), methylene diphenyl diisocyanate (MDI), and sodium montmorillonite (NaMMT) to the PLA/TPS blend to improve the compatibility between two polymers (Wang et al., 2007, 2008; Acioli-Moura and Sun, 2008; Ayana et al., 2014; Jariyasakoolroj and Chirachanchai, 2014; Xiong et al., 2014; Zuo et al., 2014; Muller et al., 2017). They all found that these compatibilizer compounds performed as a coupling agent, enhancing the interfacial interaction between the two materials, and thus improving the mechanical properties of the PLA/TPS blends. However, according to Teixeira et al. (2012), the incorporation of fibers possibly acts as a plaster in the PLA matrix because there was no major increase in tensile strength in PLA/TPS blends.

From previous studies, the PLA/Starch blend was mostly used in manufacturing biodegradable polymer and food packaging. Table 1 shows the recent studies on PLA/tarch blended applications. Starches were modified by adding a plasticizer or compatibiliser before melt blending with PLA. By doing so, the interaction between starch and the PLA matrix could be strengthened. PLA was kept pure and the common ratio of PLA and starch used in blending was 70:30, respectively (Teixeira et al., 2012).

**Table 1:** Recent studies on PLA/Starch blended applications.

**Polymers**	**Other Compounds**	**Polymer ratio**	**Processing**	**Application**	**References**
1. Sugar Palm starch 2. PLA	SPS: Glycerol (30%)	PLA:SPS 50:50	Casting/Coating	Food packaging	Sanyang et al., 2016a
1. Corn starch 2. PLA	CS: Glycerol (25%) mPEG-g-St (5–15 phr)	PLA:CS 70:30	Compression molding	Biodegradable polymer	Akrami et al., 2016
1. Corn starch 2. PLA	CS: Ester epoxidized itaconic acid (EIA) and Ether epoxidized cardanol (Epicard)	PLA/CS 70:30	Melt blending Injection molding	Biodegradable composite	Xiong et al., 2014
1. Corn starch 2. PLA	Tung oil (5–12%)	PLA:CS:TO 65:30:5 63:30:7 60:30:10 58:30:12	Melt blending Injection molding	Biodegradable polymer	Xiong et al., 2013a
1. Corn starch 2. PLA	Castor oil (5%)	PLA:CS:CO 65:30:5	Melt blending Injection molding	Biodegradable polymer	Xiong et al., 2013b

## Nanocellulose Reinforced PBS Biocomposites

**Figure 8:**
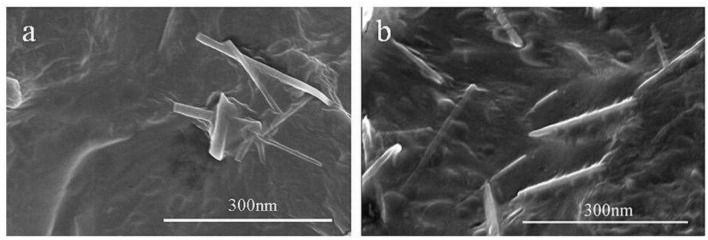
The SEM images showed **(a)** PBS/NCC **(b)** PBS/NCC/PA. The agglomeration NCC **(a)** and well-dispersed NCC **(b)** in PBS nanocomposites (adapted, with permission from Zhang and Zhang, 2015b).

Zhang and Zhang (2015) carried out a study on PBSA/CNN composites incorporated with phthalic anhydride (PA) using melt mixing and compression molding (Figure 8). Adding CNN to PBSA reduced the tensile strength and elongation at breakage as compared to pure PBSA. However, the incorporation of 2% PA increased the tensile strength by about 120% more for PBS/CNN (95:5). The water absorption of PBS/CNN (95:5) reached 1.82% after 8 h of water immersion. The addition of 2% PA into the blend composites decreased the water absorption to 0.8% with a similar immersion period. This could be related to PA's interaction with the hydroxyl groups at the PBSA and CNN surface terminals, causing low hydroxyl groups on the PBSA/PA/CNN composite surface.

**Figure 9:**
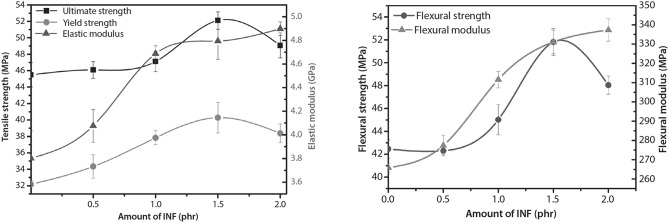
The mechanical properties of PBS/INF bionanocomposites (adapted, with permission from Joy et al., 2016).

Joy et al. (2016) prepared PBS bionanocomposites reinforced with the *Isora* nanofiber (INF) which was blended using Brabender twin-screw compounder and fabricated using an injection molding machine. Figure 9 shows that the increased quantity of (INF) in PBS/INF bionanocomposites resulted in the improvement of tensile and flexural strength due to the good distribution of the nanofiber in the PBS matrix, whereas strain at break and toughness showed the inverse results. Even so, beyond 1.5 phr of INF, the content showed a declination of tensile and flexural strength prompt to the INF agglomeration.

Zhou et al. (2016) prepared polybutylene succinate/microfibrillated cellulose (MFC) nanocomposites using the melt spinning method. MFC was first treated with acetylchloride (AC) with ball-milling to weaken the hydrogen-bonding interactions, to achieve a fine phase dispersion of MFC into the PBS matrix. The tensile strength and modulus of PBS/MFC were increased by 123 and 233%, respectively. The pretreatment of MFC was crucial since the tensile and modulus for PBS/MFC obtained through direct mixing only increased about 7 and 46%, respectively. In summary, the mechanical properties of the composite material depend on the filler dispersion, interfacial bonding of the matrix and filler, distribution of filler, and the matrix molecular chain.

Li et al. (2017) studied the morphology, rheological, and crystallization behavior of PBS/NCC bionanocomposites fabricated using the solution coagulation method. The SEM images displayed a well-dispersed NCC in the PBS matrix and rheological analysis indicated a strong interfacial bonding between PBS and NCC. The glass transition temperature was increased slightly and improved in the crystallization temperature and the various melting behaviors of the bionanocomposites as compared to pure PBS. Small loading of NCC resulted in increment of the overall crystallization rate of bionanocomposites and kept the crystal structure and crystallization mechanism unchanged. Overall improvement in the crystallization behavior of the bionanocomposites could be associated with the exceptional nucleation ability of well-dispersed NCC nano-sized particles.

Xu et al. (2019) conducted a study on the mechanical, morphological, and barrier properties of PBS reinforced with chitin whiskers and nanocrystalline cellulose film using a hot press. By using transmission electron microscopy (TEM), the dispersion of nanofillers could be seen in the polymer matrix, preventing the movement of polymer chains, nurturing the recrystallisation of polymers, causing an increment in the degree of crystallinity from 65.9 to 75.6%. The Incorporation of NCC and CW into PBS nanocomposite films increased the tensile strength from 23.2 to 32.9 and 43.6 MPa, respectively. Adding 3% of NCC resulted in rapidly dropped OTR from 737.7 to 280 cc/m^2^/day, and by inserting 4% of MDI further decreased to 23.8 cc/m^2^/day. The incorporation of 3% NCC reduced the WVTR from 83.8 to 49.4 g/m^2^/day, and 4% of MDI further decreased to 30.8 g/m^2^/day.

### PBS/Starch Blend Biocomposites

Compared to conventional plastics, PBS is still expensive and lacks properties required for high-end use, such as gas barrier properties, softness, and melting viscosity. It is therefore often mixed with natural biopolymers, such as cellulose, starch, soy protein, and various plant fibers to reduce the cost of production and to promote different applications. Starch is well-known for its usage as an additive in biodegradable polymers because of its high biodegradation capability. Besides its abundancy, affordability, renewability, and biodegradability, starch needs to be modified with plasticizers to decrease the intermolecular interactions and to increase its processability (Liu et al., 2015). Starch is often blended with other materials due to low physical properties and processing capability. It is also bended to improve the limitation properties of both polymers which produce a superior material for various end applications. However, this interfacial bonding is crucial for excellent thermal and mechanical performances (Zhang et al., 2019a). Since both polymers are thermodynamically immiscible, compatibilizer incorporation and starch modification should be employed to improve the interfacial miscibility for desirable performances.

Blending natural polymers withaliphatic polyesters can improve the overall mechanical and water resistance properties without negatively affecting their biodegradability (Garalde et al., 2019). The major problem is their compatibility due to hydrophilicity of starch and hydrophobicity of PBS, resulting in poor adhesion bonding between the two materials (Zeng et al., 2011). Starch is a bio-based polymer that is widely used in medical applications, construction, automotive, and consumer goods and packaging (Hemamalini et al., 2018). Starch plasticization is the method of breaking the strong inter-molecular and intra-molecular hydrogen bonds using plasticizer (mainly glycerol), heat, and shear to improve the processability. However, after the modification of starch, the PBS/TPS blend still requires a compatibilizer to act as a coupling agent to develop efficient physical and chemical reactions to form stronger interfacial bonding and a dispersed phase size (Suchao-In et al., 2014). The essential factor which encourages the decomposition of polymers is a moist environment. Microorganisms thrive in humid surroundings to break down the polymers at the end of their life. In some practices, adding natural fiber is vital in accelerating the decomposition of polymers (Calabia et al., 2013). Table 2 shows the water barrier properties of the PBS based composite incorporated with starch and nanocellulose. Thus, it is solely dependent on the application of the polymer materials to attain desirable properties by infusing it with various proportions of filler.

**Table 2:** Water barrier properties of PBS based composite incorporated with starch and nanocellulose.

**Polymer**	**Plasticizer/Compatibilizer/Filler**	**Description**	**References**
Corn starch PBS	Glycerol	• Increasing value of RPBS in the sample decreases the water absorption. TPS water absorption. was more than 300% and as RPBS was increased to 50%, the water absorption decreases to 21.2%.	Zeng et al., 2011
Cassava starch PBSL	Luffa fiber (LF) Kenaf fiber (KF)	• Increasing starch value in the sample increases the water absorption. • The water absorption was further increased with the addition of LF and KF with LF incorporated composite reached up to 5 wt.%. This is due to the hydrophilic feature and porous structure of those plant fibers.	Lai et al., 2016
Cassava starch PBS	Maleic anhydride Cloisite30B CloisiteNa	• Both Cloisite30B and CloisiteNa decrease OTR and WVTP of PBS/TPS (75:25). WVTR was further decreased using Cloisite30B compared to CloisiteNa suggesting that CloisiteNa is more hydrophilic than Cloisite30B.	Boonprasith et al., 2013
Cassava starch PBS	Glycerol Maleic anhydride (rPBS)	• The samples containing rPBS have lower equilibrium water uptake compared to the sample without one. The water molecules diffusion was restricted by the homogeneous microstructure and good dispersion of starch with PBS. The quantity of compatibilizer does not influence the sample's water resistance.	Yin et al., 2015
Corn starch (Waxy and normal starch) PBS	Glycerol	• WTPS has higher water absorption than NTPS PBS/WTPS blends have slightly lower water absorption compared to PBS/NTPS blends. WTPS particles are finely dispersed in the PBS matrix due to its low melt viscosity.	Li et al., 2013
Corn flour PBSA	Glycerol	• The PBSA/TPS blend (70:30) hydration rate was almost identical to pure PBSA.	Jbilou et al., 2013
PBS	Glycerol MDI NCC Chitin whiskers (CW)	• OTR and WVTR of PBS/NCC (97:3) films were reduced by 62% and 41% respectively as compared to neat PBS films. Adding MDI further reduced OTR and WVTR of PBS/NCC (97:3) films by 92% and 38%, respectively.	Xu et al., 2019
PBSA	Phthalic anhydride (PA) NCC	• The water absorption of PBSA/NCC reaches 1.82% after 8 h of being immersed in water. Adding PA into PBSA/NCC reduced the water absorption to 0.8% at the same immersion time. Increasing the value of NCC in PBSA/NCC up to 10% increases the permeability rate but decreases with further addition.	Zhang and Zhang, 2015

## Conclusions

The incorporation of nanocellulose is indeed beneficial to enhance the practical properties of TPS, PLA, and PBS for food packaging. TPS/nanocellulose improved their low water barrier and tensile properties. The loading of nanocellulose into PLA and PBS improved their oxygen barrier and mechanical properties. Higher loading of nanocellulose did not necessarily increase their practical properties. Agglomeration occurred if loading of nanocellulose in the polymers was exceeded. Compatibilizer was used to promote fine dispersion and to form strong interfacial bonding of fillers into the polymer matrix. This leads to stronger mechanical and barrier properties with biodegradable properties. In food packaging, plastics are single-use but must have decent mechanical and barrier properties. Nanocellulose reinforced polymers can be potentially used in food packaging applications.

## Author Contributions

SSa: introduction and checking the content of manuscript. AN: idea and write up of biopolymer. RI: starch and nanocellulose. SSh: PLA bionanocomposites. RS: PBS bionanocomposites. MZ: proofread the manuscript.

### Conflict of Interest

The authors declare that the research was conducted in the absence of any commercial or financial relationships that could be construed as a potential conflict of interest.
